# Inhibition of abnormal C/EBPβ/α‐Syn signaling pathway through activation of Nrf2 ameliorates Parkinson's disease‐like pathology

**DOI:** 10.1111/acel.13958

**Published:** 2023-08-23

**Authors:** Zefang Lin, Lixuan Huang, Qianqian Cao, Hanyue Luo, Wei Yao, Ji‐chun Zhang

**Affiliations:** ^1^ Department of Physiology, School of Medicine Jinan University Guangzhou China; ^2^ Guangzhou Key Laboratory of Formula‐pattern Research Center, School of Traditional Chinese Medicine Jinan University Guangzhou China; ^3^ Institute of Brain Science and Brain‐inspired Research Shandong First Medical University & Shandong Academy of Medical Sciences Jinan China

**Keywords:** C/EBPβ, Nrf2, Parkinson's disease, transcription, α‐Syn

## Abstract

Parkinson's disease (PD) is characterized by the formation of Lewy bodies (LBs) in the brain. These LBs are primarily composed of α‐Synuclein (α‐Syn), which has aggregated. A recent report proposes that CCAAT/enhancer‐binding proteins β (C/EBPβ) may act as an age‐dependent transcription factor for α‐Syn, thereby initiating PD pathologies by regulating its transcription. Potential therapeutic approaches to address PD could involve targeting the regulation of α‐Syn by C/EBPβ. This study has revealed that Nrf2, also known as nuclear factor (erythroid‐derived 2)‐like 2 (NFE2L2), suppresses the transcription of C/EBPβ in SH‐SY5Y cells when treated with MPP^+^. To activate Nrf2, sulforaphane, an Nrf2 activator, was administered. Additionally, C/EBPβ was silenced using C/EBPβ‐DNA/RNA heteroduplex oligonucleotide (HDO). Both approaches successfully reduced abnormal α‐Syn expression in primary neurons treated with MPP^+^. Furthermore, sustained activation of Nrf2 via its activator or inhibition of C/EBPβ using C/EBPβ‐HDO resulted in a reduction of aberrant α‐Syn expression, thus leading to an improvement in the degeneration of dopaminergic neurons in the substantia nigra pars compacta (SNc) in mouse models induced by 1‐methyl‐4‐phenyl‐1,2,5,6‐tetrahydropyridine (MPTP) and those treated with preformed fibrils (PFFs). The data presented in this study illustrate that the activation of Nrf2 may provide a potential therapeutic strategy for PD by inhibiting the abnormal C/EBPβ/α‐Syn signaling pathway.

## Graphical abstract

CCAAT/enhancer‐binding protein β (C/EBPβ) is an age‐dependent transcription factor that regulates the expression of α‐Synuclein (α‐Syn) in the brain of Parkinson's disease (PD) mouse mode. Sulforaphane (SFN) can activate Nrf2, which inhibits abnormal a‐Syn expression by inhibiting C/EBPß. Moreover, C/EBPß‐HDO can silence the expression of C/EBPß, resulting in a similar inhibition of a‐Syn expression.

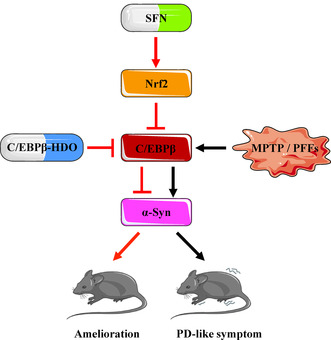

**Abbreviations**
AAV: adeno‐associated virus
AD: Alzheimer's disease
AREs: antioxidant response elements
ASOs: antisense oligonucleotides
C/EBPβ: CCAAT/enhancer binding proteins β
ChIP: chromatin immunoprecipitation
ELISA: enzyme‐linked immunosorbent assay
HDO: DNA/RNA heteroduplex oligonucleotide
LBs: Lewy bodies
LNAs: locked nucleic acids
MPP+: 1‐methyl‐4‐phenylpyridinium
MPTP: 1‐methyl‐4‐phenyl‐1,2,5,6‐tetrahydropyridine
Nrf2: nuclear factor (erythroid‐derived 2)‐like 2
PD: Parkinson's disease
PFA: paraformaldehyde
PFFs: preformed fibrils
PVDF: polyvinylidene difluoride
qPCR: quantitative PCR
SFN: sulforaphane
SNc: substantia nigra pars compacta
TBS: tris‐buffered saline
α‐Syn: α‐Synuclein

## 1 INTRODUCTION

Neurodegeneration in Parkinson's disease (PD) is marked by the progressive deterioration of dopaminergic neurons in the substantia nigra pars compacta (SNc). This results in motor symptoms as a consequence of the simultaneous loss of nigrostriatal dopaminergic termini. PD is the second most prevalent neurodegenerative disorder that occurs with age (Kang, Zhang, Liu, Manfredsson, He, et al., 2017). The etiology of PD is presently unclear. However, it is noteworthy that both sporadic and familial PD share identical pathological characteristics. Specifically, degeneration of dopaminergic neurons occurs in the SNc, with the remaining dopaminergic neurons containing intraneuronal proteinaceous cytoplasmic inclusions referred to as Lewy bodies (LBs). These pathological features are commonly observed in both sporadic and familial cases of PD (Dauer & Przedborski, 2003; Yang et al., 2020). The main component of LBs is alpha‐synuclein (α‐Syn), and the aggregation of α‐Syn is considered a critical factor in the development of PD (Volpicelli‐Daley et al., 2011). Phosphorylation of the α‐Syn protein at the serine 129 site is a critically important post‐translational modification that enhances the generation of pathogenic α‐Syn aggregates (Zhang et al., 2019). The pathogenesis of both sporadic and familial PD has been identified as the mutation or multiplication of α‐Syn (Ellis et al., 2001; Jiang et al., 2013; Okochi et al., 2000). Mutations in the α‐Syn (SNCA) gene, including A53T, A30P, E46K, H50Q, Q51D, and A53E, lead to autosomal‐dominant PD (Toffoli et al., 2020). Soluble monomers, toxic oligomers, and insoluble fibrils of α‐Syn have been observed in the brains of patients diagnosed with PD (Lashuel et al., 2013). These findings suggest that the abnormal accumulation of α‐Syn contributes to the accelerated death of dopaminergic neurons in both familial and sporadic cases of PD.

CCAAT/enhancer‐binding proteins (C/EBP) belong to the class of transcription factors known as basic‐leucine zipper (bZIP). The family consists of six members, with C/EBPβ being particularly abundant in the brain. In this context, it plays a crucial role in memory formation and synaptic plasticity (Pulido‐Salgado et al., 2015). C/EBPβ can be activated by pro‐inflammatory cytokines such as IL‐1β, IL‐6, and TNF‐α. It regulates the pro‐inflammatory program in glial cells, playing a crucial role in inducing neurotoxic effects through microglia activation (Magalini et al., 1995; Poli, 1998; Pulido‐Salgado et al., 2015; Straccia et al., 2011; Wedel & Ziegler‐Heitbrock, 1995). It has been reported that the expression of C/EBPβ in human neuroblastoma cells increases with dopamine treatment in a dose‐dependent manner. This increase in expression is correlated with the sequential escalation of α‐Syn, indicating that C/EBPβ may be involved in the development of PD (Gomez‐Santos et al., 2005). Recent studies have confirmed the link between C/EBPβ and the onset of neurodegenerative disorders. C/EBPβ acts as a key transcription factor for δ‐secretase and regulates its mRNA levels in both aging and Alzheimer's disease (AD) brains (Wang et al., 2018). Moreover, C/EBPβ serves as the primary transcription factor for α‐Syn and regulates their mRNA expressions during aging and in the brains of individuals affected by PD (Wu et al., 2021). As a result, inhibiting abnormal C/EBPβ expression could be a potential therapeutic approach to counteract the advancement of PD.

Nrf2 (nuclear factor (erythroid‐derived 2)‐like 2; NFE2L2) is a transcription factor that plays a critical role in mediating the expression of Phase II detoxification and antioxidant genes. Upon activation, Nrf2 disassociates from Keap1 and translocates to the nucleus where it dimerizes with other bZIP proteins, such as small Maf proteins. This process forms a transactivation complex that binds to antioxidant response elements (AREs), enabling the transactivation of target genes involved in cellular defense against oxidative stress (Kobayashi et al., 2013; Kometsi et al., 2020; Ma, 2013; Suzuki et al., 2013; Suzuki & Yamamoto, 2015; Yamamoto et al., 2018). Sulforaphane (SFN), an isothiocyanate compound naturally occurring in broccoli, has been discovered as a potent activator of the Nrf2 signaling pathway. Activation of this pathway by SFN results in enhanced antioxidant and anti‐inflammatory effects (Cheung & Kong, 2010; Fahey et al., 1997; Lin et al., 2008; Zhang et al., 1994). Since Nrf2 plays a crucial role as a transcriptional mediator of antioxidants, it is worth investigating its potential to inhibit C/EBPβ transcription. Pro‐inflammatory cytokines are known to activate C/EBPβ, which in turn regulates α‐Syn expression in both aging and PD brains. Therefore, exploring the effects of Nrf2 activation on the abnormal C/EBPβ/α‐Syn signaling pathway in a PD mouse model could provide valuable insights into mitigating PD‐like pathology.

## 2 RESULTS

### 2.1 The activation of Nrf2 by SFN and the overexpression of Nrf2 inhibit the transcription of C/EBPβ in SH‐SY5Y cells treated with 1‐methyl‐4‐phenylpyridinium (MPP ^+^)

**FIGURE 1:**
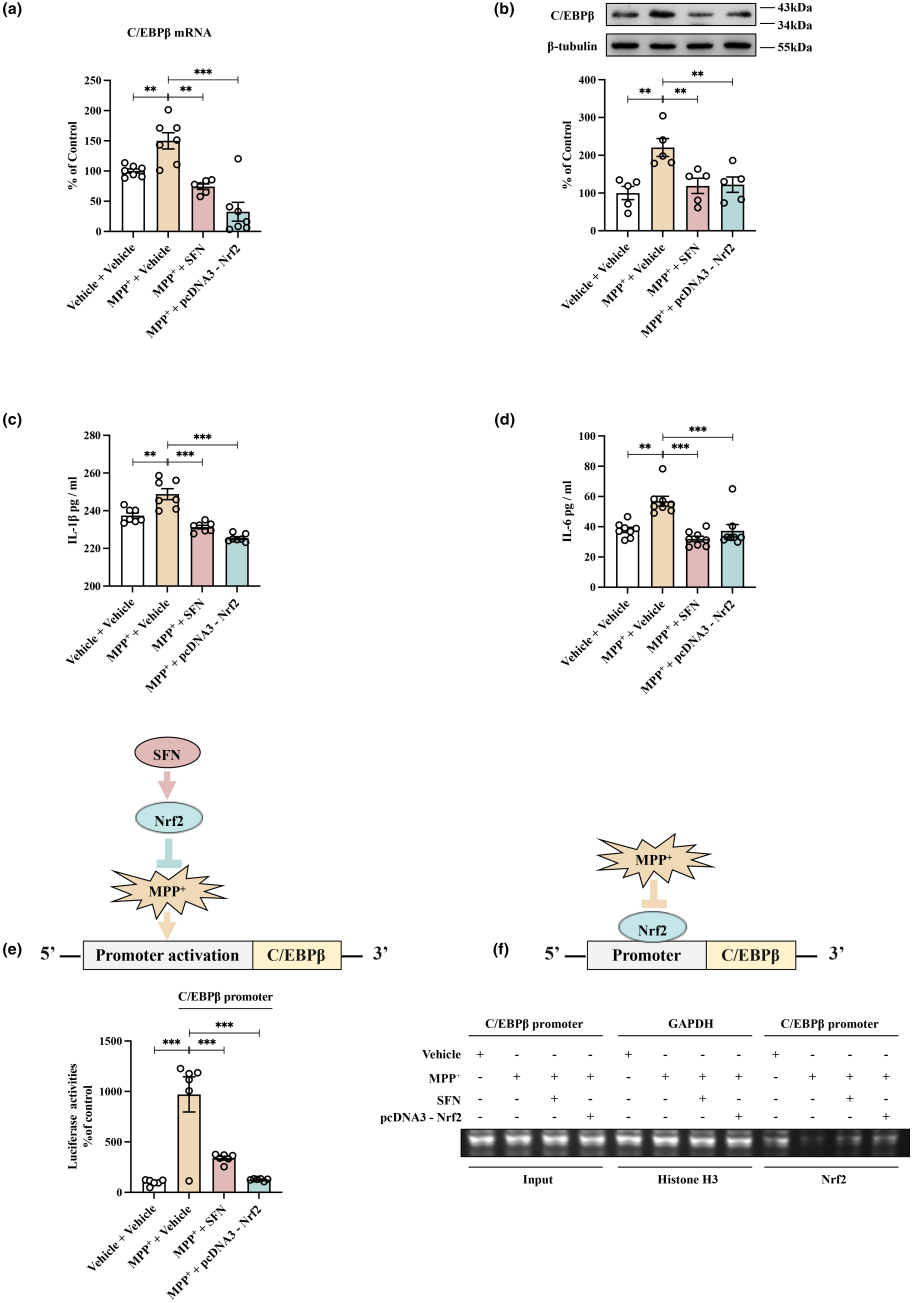
Activating Nrf2 using SFN or overexpressing Nrf2 can effectively suppress C/EBPβ transcription. (a,b) Treatment with SFN or Nrf2 overexpression effectively mitigates the induction of C/EBPβ mRNA and protein levels in SH‐SY5Y cells stimulated with MPP^+^. Both qPCR and western blot analyses provide compelling evidence showing significant decreases in C/EBPβ mRNA and protein levels following SFN or Nrf2 treatments, as compared to MPP^+^ alone‐treated cells (mean ± SEM, *n* = 6 or 7 per group for mRNA, *n* = 5 per group for protein, one‐way ANOVA, **p < 0.01, ***p < 0.001). (c, d) Treatments with SFN or Nrf2 overexpression are highly effective in suppressing the induction of pro‐inflammatory cytokine expressions triggered by MPP^+^ in SH‐SY5Y cells. The levels of IL‐1β and IL‐6 noticeably reduced following treatment with either SFN or Nrf2, as compared to the MPP^+^ treated group, as demonstrated by ELISA assay results (mean ± SEM, *n* = 7 or 8 per group, one‐way ANOVA, **p < 0.01, ***p < 0.001). (e) Treatment with SFN or Nrf2 overexpression effectively suppresses MPP^+^‐induced C/EBPβ promoter activation. The luciferase reporter assay results show remarkable reductions in C/EBPβ promoter activity levels following treatment with either SFN or Nrf2, as compared to MPP^+^ treated cells (mean ± SEM, *n* = 6 per group, one‐way ANOVA, ***p < 0.001). (f) Treatment with SFN or Nrf2 overexpression can effectively mitigate the MPP^+^‐induced dissociative effects of Nrf2 with the C/EBPβ promoter in SH‐SY5Y cells. ChIP assay results demonstrate an increase in the dissociation of Nrf2 with the C/EBPβ promoter following treatment with MPP^+^, which is blocked upon treatment with either SFN or Nrf2 overexpression.

A recent study has demonstrated the critical role of C/EBPβ as a transcription factor for α‐Syn, highlighting its involvement in regulating α‐Syn expression in both aging and PD brains (Wu et al., 2021). In this study, our objective was to examine the potential of Nrf2 activator SFN and Nrf2 overexpression in inhibiting the transcription of the C/EBPβ gene in SH‐SY5Y cells treated with MPP^+^. Initially, we utilized quantitative PCR (qPCR) and western blot assays to assess the impact of MPP^+^ on mRNA and protein expression of C/EBPβ in SH‐SY5Y cells. The results revealed that MPP^+^ significantly induced an elevation in both C/EBPβ mRNA and protein levels. However, this effect was effectively abrogated by treatment with SFN and overexpression of Nrf2 (Figure 1a,b). Secondly, it is of interest to investigate the expression of pro‐inflammatory cytokines IL‐1β and IL‐6 in MPP^+^‐treated SH‐SY5Y cells, as these cytokines are known to activate C/EBPβ and induce neurotoxic effects (Magalini et al., 1995; Poli, 1998; Pulido‐Salgado et al., 2015; Straccia et al., 2011; Wedel & Ziegler‐Heitbrock, 1995). Enzyme‐linked immunosorbent assay (ELISA) revealed that the levels of IL‐1β and IL‐6 were increased in SH‐SY5Y cells treated with MPP^+^, but were subsequently reduced following treatment with SFN and Nrf2 overexpression (Figure 1c,d). Finally, to further clarify the impact of SFN and Nrf2 overexpression on inhibiting the expression of C/EBPβ, we conducted luciferase reporter assays and a chromatin immunoprecipitation (ChIP) assay. The luciferase reporter assays revealed that MPP^+^ activated the C/EBPβ promoter in SH‐SY5Y cells; however, this activation was inhibited by SFN and Nrf2 overexpression (Figure 1e). Furthermore, as depicted in Figure 1f, our ChIP assay indicated that MPP^+^ increased the dissociation effects of Nrf2 from the C/EBPβ promoter in SH‐SY5Y cells; nevertheless, this effect was relieved by SFN and Nrf2 overexpression. The results of this study suggest that both SFN and Nrf2 overexpression exert a protective effect against MPP^+^‐induced damage in SH‐SY5Y cells by reducing the transcription of C/EBPβ.

### 2.2 The activation of Nrf2 by SFN or the silencing of C/EBPβ expression by C/EBPβ‐DNA/RNA heteroduplex oligonucleotide (HDO) can reduce abnormal α‐Syn expression in primary neurons treated with MPP^+^

**FIGURE 2:**
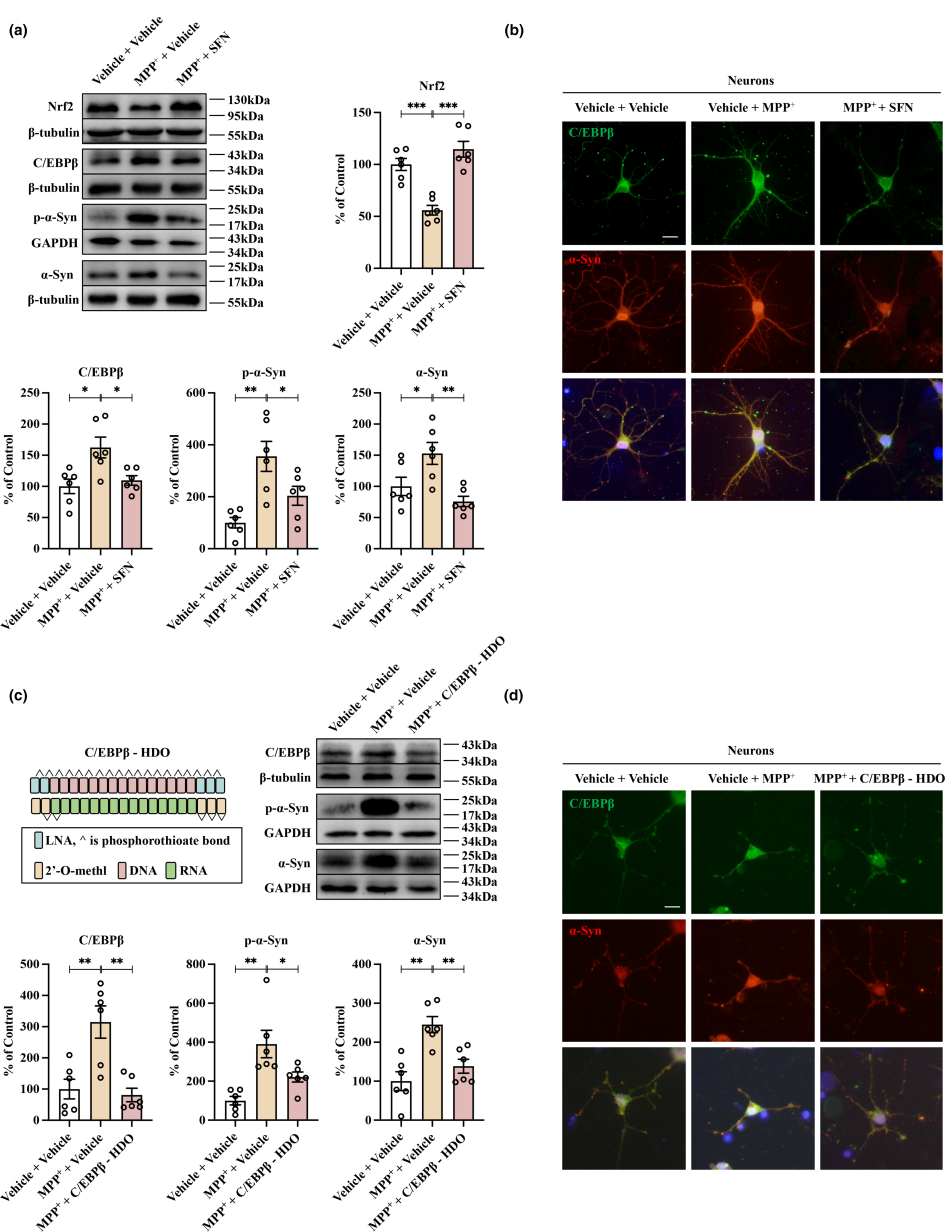
Treating primary neurons with SFN to activate Nrf2 or using C/EBPβ‐HDO to silence C/EBPβ expression can effectively mitigate the upregulation of abnormal α‐Syn expression triggered by MPP^+^. (a) SFN significantly prevents the abnormalities caused by MPP^+^ in primary neurons, including the downregulation of Nrf2 and the upregulation of C/EBPβ, p‐α‐Syn, and α‐Syn expression levels. This attenuation was confirmed using western blot analysis (mean ± SEM, *n* = 6 per group, one‐way ANOVA, *p < 0.05, **p < 0.01, and ***p < 0.001). (b) Immunofluorescence staining analysis shows that treatment with SFN can effectively reduce the levels of C/EBPβ and α‐Syn expression in primary neurons treated with MPP^+^. Scale bar = 50 μm. (c) Schematic illustration of the construction of C/EBPβ‐HDO. C/EBPβ‐HDO administration is an effective approach to reducing the upregulation of both C/EBPβ, p‐α‐Syn, and α‐Syn expression levels in MPP^+^‐treated primary neurons. This was confirmed using western blot analysis (mean ± SEM, *n* = 6 per group, one‐way ANOVA, *p < 0.05, **p < 0.01). (d) C/EBPβ‐HDO treatment leads to a significant decrease in both C/EBPβ and α‐Syn immunoreactivity levels in primary neurons that have been treated with MPP^+^. Scale bar = 50 μm.

SFN‐induced activation of Nrf2 reduces transcription of C/EBPβ in vitro. C/EBPβ, the primary transcription factor controlling α‐Syn expression in PD, can potentially be regulated through activation of Nrf2. Therefore, we investigated whether SFN‐induced activation of Nrf2 could decrease abnormal α‐Syn expression by inhibiting the expression of C/EBPβ in vitro. To investigate this hypothesis, primary neurons were treated with MPP^+^ to establish a model. The results revealed a significant reduction in Nrf2 expression and an increase in C/EBPβ, p‐α‐Syn, and α‐Syn expression in primary neurons following MPP^+^ treatment (Figure 2a). However, the administration of SFN reversed these effects (Figure 2a). Immunofluorescence staining further demonstrated that MPP^+^ caused a redistribution of both C/EBPβ and α‐Syn within primary neurons, resulting in elevated immunoreactivity (Figure 2b). Again, SFN treatment effectively reversed this effect (Figure 2b). Additionally, nuclear/cytosol fractionation immunoblot analysis indicated that C/EBPβ was predominantly expressed in the nuclei of primary neurons. MPP^+^ treatment increased C/EBPβ expression, which was mitigated by SFN (Figure S1).

Subsequently, we developed C/EBPβ‐HDO, a gene silencing technology designed to suppress the expression of C/EBPβ (Asada et al., 2021; Nishina et al., 2015; Yoshioka et al., 2019). The effectiveness of C/EBPβ‐HDO in suppressing C/EBPβ mRNA and protein levels in SH‐SY5Y cells was confirmed through qPCR and western blot experiments (Figure S2A,B). These results indicate that C/EBPβ‐HDO holds promise as a tool for inhibiting C/EBPβ expression. To further explore the potential of C/EBPβ‐HDO, we investigated whether inhibiting C/EBPβ expression could alleviate abnormal α‐Syn expression in primary neurons treated with MPP^+^. Western blot analysis revealed a significant increase in the expression of C/EBPβ and α‐Syn in MPP^+^‐treated primary neurons (Figure 2c). However, this effect was reversed with the use of C/EBPβ‐HDO (Figure 2c). Immunofluorescence staining also showed an increase in the immunoreactivity of C/EBPβ and α‐Syn in MPP^+^‐treated primary neurons, which was again attenuated by C/EBPβ‐HDO (Figure 2d). Furthermore, nuclear/cytosol fractionation immunoblot analysis indicated that C/EBPβ‐HDO reduced the elevated C/EBPβ expression in the nucleus of MPP^+^‐treated primary neurons (Figure S3). The results demonstrated that the activation of Nrf2 or the silencing of C/EBPβ expression was effective in decreasing aberrant α‐Syn expression in primary neurons treated with MPP^+^.

### 2.3 Activation of Nrf2 by glucoraphanin has been demonstrated to decrease the degeneration of dopaminergic neurons in A53T mice treated with 1‐methyl‐4‐phenyl‐1,2,5,6‐tetrahydropyridine (MPTP)

**FIGURE 3:**
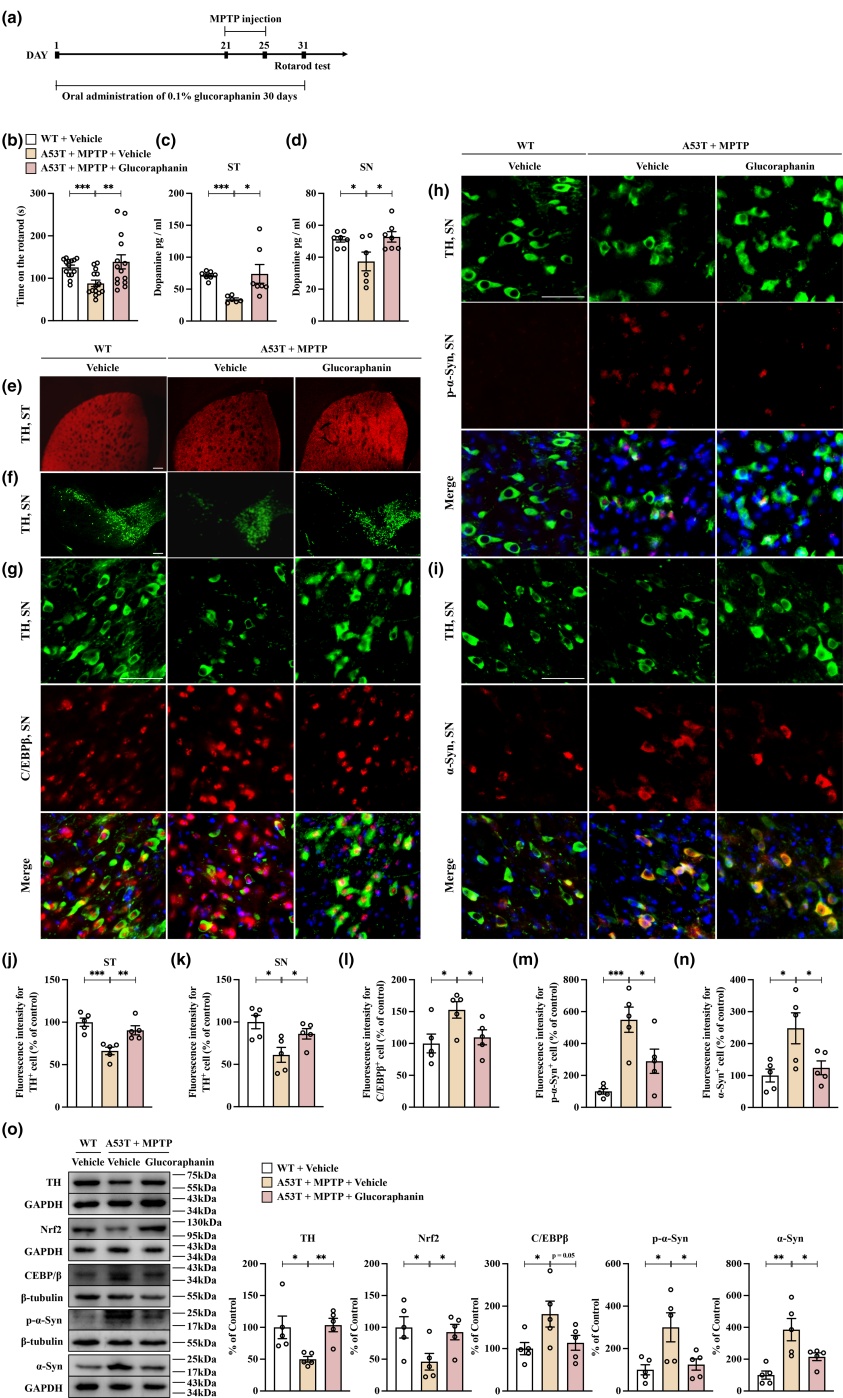
Activating Nrf2 can significantly mitigate the degeneration of dopaminergic neurons in A53T mice treated with MPTP. (a) Schedule of treatment. (b) Rotarod test. The duration time for the rotarod test were recorded and compared (mean ± SEM, *n* = 14 per group, one‐way ANOVA, **p < 0.01 and ***p < 0.001). (c, d) Glucoraphanin attenuated the decreased concentration of dopamine in the striatum and SNc of MPTP‐treated A53T mice. ELISA assay showed that the decreased concentration of dopamine in the striatum and SNc of MPTP‐treated A53T mice were significantly ameliorated by glucoraphanin (mean ± SEM, *n* = 6 or 7 per group, one‐way ANOVA, *p < 0.05, ***p < 0.001). (e,f) Immunofluorescence staining showed that the TH neuron reduction in both striatum and SNc regions with the treatment of MPTP, which was attenuated by treatment with glucoraphanin. Scale bar = 200 μm. (g) Immunofluorescent double staining of TH and C/EBPβ demonstrates that the loss of TH neurons is coupled with an increase of C/EBPβ. Scale bar = 50 μm. (h) Immunofluorescent double staining of TH and p‐α‐Syn demonstrates that the loss of TH neurons is coupled with an increase of p‐α‐Syn. Scale bar = 50 μm. (i) Immunofluorescent double staining of TH and α‐Syn demonstrates that the loss of TH neurons is coupled with an increase of α‐Syn. Scale bar = 50 μm. (j‐n) The immunostaining intensities of TH, C/EBPβ, p‐α‐Syn, and α‐Syn were measured and compared among (mean ± SEM, *n* = 5 per group for TH, C/EBPβ, p‐α‐Syn, and α‐Syn, one‐way ANOVA, *p < 0.05, **p < 0.01, and ***p < 0.001). (o) Glucoraphanin attenuates the downregulation of TH and Nrf2 and the upregulation of C/EBPβ, p‐α‐Syn, and α‐Syn expression in MPTP‐treated A53T mice. Western blot suggested that administration of glucoraphanin significantly ameliorated the downregulation of Nrf2 and TH and the upregulation of C/EBPβ, p‐α‐Syn, and α‐Syn protein expression in the SNc of MPTP‐treated A53T mice (mean ± SEM, *n* = 5 per group, one‐way ANOVA, *p < 0.05, **p < 0.01).

MPTP is a well‐known neurotoxin that is responsible for inducing pathology similar to PD. Previous studies have demonstrated that injecting MPTP in SNCA mice can lead to the progression of PD (Kang, Zhang, Liu, Manfredsson, Benskey, et al., 2017; Luo et al., 2019). Our in vitro findings suggest that activation of Nrf2 by SFN inhibits the transcription of C/EBPβ, thus preventing abnormal expression of α‐Syn. In this study, we aimed to determine whether Nrf2 activation can alleviate MPTP‐induced PD‐like symptoms in mice by suppressing C/EBPβ expression. To test this hypothesis, we used glucoraphanin, an activator of Nrf2 and a biosynthetic precursor of SFN (Yao et al., 2016). In a 30‐day oral administration of 0.1% glucoraphanin, we observed a significant increase in the duration time of the rotarod test for A53T mice treated with MPTP (Figure 3a,b). Additionally, the ELISA assay showed that glucoraphanin treatment effectively improved the reduced dopamine concentration in the striatum and SNc of A53T mice treated with MPTP (Figure 3c,d). Furthermore, immunofluorescence staining revealed that glucoraphanin attenuated the decreased TH immunoreactivity in both the striatum and SNc of MPTP‐treated A53T mice (Figure 3e,f,j,k). Co‐staining of TH with C/EBPβ, p‐α‐Syn, and α‐Syn proteins suggested that the loss of TH neurons in the SNc of MPTP‐treated A53T mice is associated with an increase in C/EBPβ, p‐α‐Syn, and α‐Syn levels, but these effects were markedly reduced after glucoraphanin treatment (Figure 3g‐i, l‐n). Immunofluorescent staining for IBA1 and GFAP also found that glucoraphanin significantly alleviated the elevated IBA1 and GFAP immunoreactivity in the SNc of MPTP‐treated A53T mice (Figure S4). Western blot analysis further confirmed the beneficial effects of glucoraphanin on TH and Nrf2 protein expression, while decreasing the levels of C/EBPβ, p‐α‐Syn, and α‐Syn protein expression in the SNc of MPTP‐treated A53T mice (Figure 3o). According to the results, glucoraphanin has been shown to improve PD‐like symptoms in A53T mice treated with MPTP. This suggests that the activation of Nrf2 through glucoraphanin leads to the inhibition of abnormal expression of C/EBPβ and α‐Syn in the SNc of MPTP‐treated A53T mice, resulting in a reduction in the degeneration of dopaminergic neurons.

### 2.4 Silencing the expression of C/EBPβ using C/EBPβ‐HDO has been demonstrated to mitigate the degeneration of dopaminergic neurons in A53T mice when treated with MPTP

**FIGURE 4:**
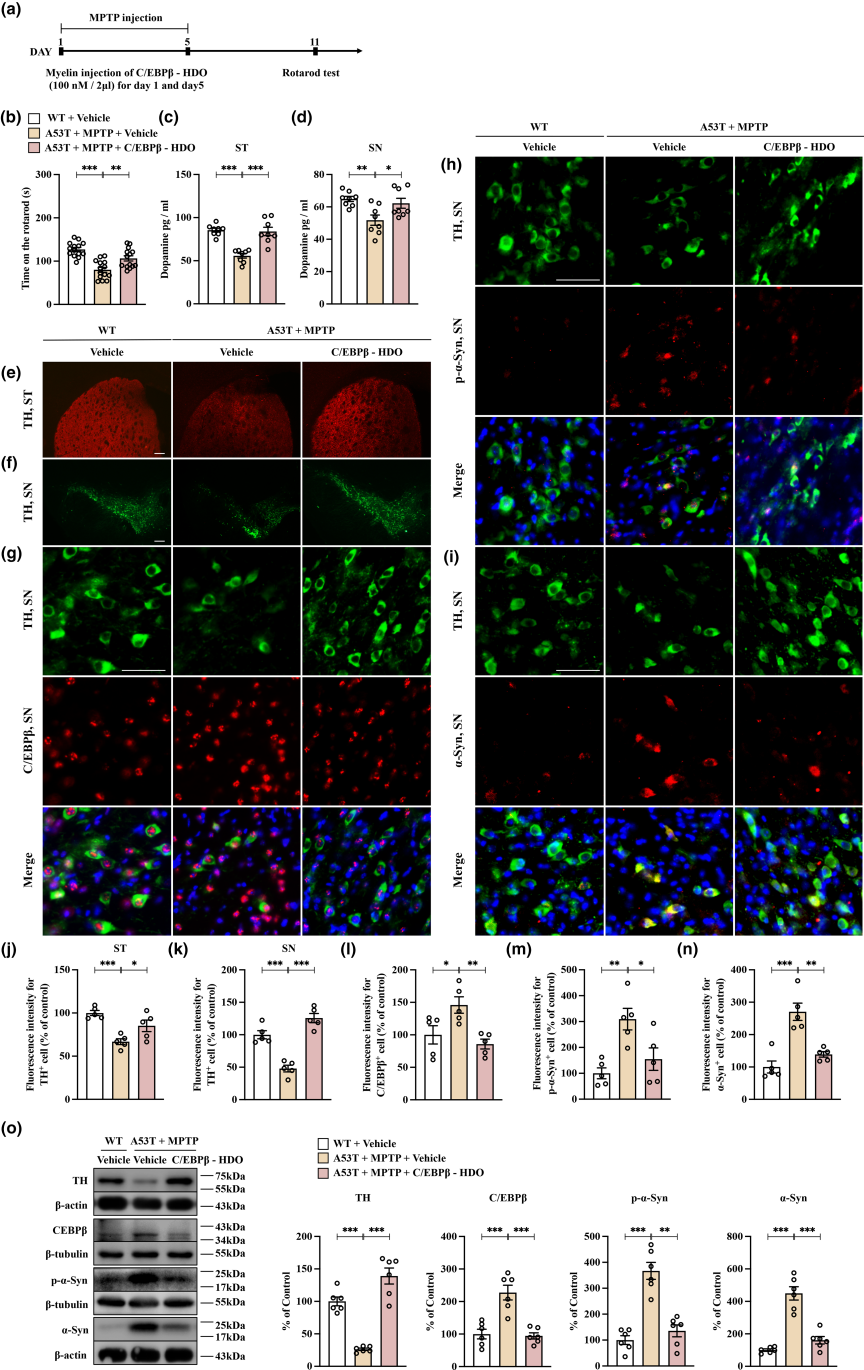
Silencing the expression of C/EBPβ can significantly reduce the degeneration of dopaminergic neurons in A53T mice treated with MPTP. (a) Schedule of treatment. (b) Rotarod test. The duration time for the rotarod test were recorded and compared (mean ± SEM, *n* = 14 per group, one‐way ANOVA, **p < 0.01 and ***p < 0.001). (c, d) C/EBPβ‐HDO attenuated the decreased concentration of dopamine in the striatum and SNc of MPTP‐treated A53T mice. ELISA assay showed that the decreased concentration of dopamine in the striatum and SNc of MPTP‐treated A53T mice were significantly ameliorated by C/EBPβ‐HDO (mean ± SEM, *n* = 8 per group, one‐way ANOVA, *p < 0.05, **p < 0.01, and ***p < 0.001). (e,f) Immunofluorescence staining showed the TH neuron reduction in both striatum and SNc regions with the treatment of MPTP, which was attenuated by treatment with C/EBPβ‐HDO. Scale bar = 200 μm. (g) Immunofluorescent double staining of TH and C/EBPβ demonstrates that the loss of TH neurons is coupled with an increase of C/EBPβ. Scale bar = 50 μm. (h) Immunofluorescent double staining of TH and p‐α‐Syn demonstrates that the loss of TH neurons is coupled with an increase of p‐α‐Syn. Scale bar = 50 μm. (i) Immunofluorescent double staining of TH and α‐Syn demonstrates that the loss of TH neurons is coupled with an increase of α‐Syn. Scale bar = 50 μm. (j‐n) The immunostaining intensities of TH, C/EBPβ, p‐α‐Syn, and α‐Syn were measured and compared among (mean ± SEM, *n* = 5 per group for TH, C/EBPβ, p‐α‐Syn, and α‐Syn, one‐way ANOVA, *p < 0.05, **p < 0.01, and ***p < 0.001). (o) C/EBPβ‐HDO attenuates the downregulation of TH and Nrf2 and the upregulation of C/EBPβ, p‐α‐Syn, and α‐Syn expression in MPTP‐treated A53T mice. Western blot suggested that administration of C/EBPβ‐HDO significantly ameliorated the downregulation of Nrf2 and TH and the upregulation of C/EBPβ, p‐α‐Syn, and α‐Syn protein expression in the SNc of MPTP‐treated A53T mice (mean ± SEM, *n* = 6 per group, one‐way ANOVA, **p < 0.01 and ***p < 0.001).

The activation of Nrf2 by its activator inhibits the abnormal expression of both C/EBPβ and α‐Syn in the SNc of A53T mice treated with MPTP. This study employed a C/EBPβ‐HDO construct to investigate the direct silencing effect on C/EBPβ expression in A53T mice treated with MPTP, with the aim of analyzing its impact on abnormal α‐Syn expression. The mice received a total of four intrathecal injections of C/EBPβ‐HDO at a concentration of 100 nM, with a volume of 2 μL per week. In the SNc of wild‐type (WT) mice, C/EBPβ‐HDO significantly decreased both C/EBPβ mRNA and protein expression levels (Figure S5A,B). Subsequently, in A53T mice treated with MPTP, two repeated intrathecal administrations of C/EBPβ‐HDO (100 nM per 2 μL) on Day 1 and Day 5 significantly improved the duration time in the rotarod test (Figure 4a,b). ELISA analysis revealed that C/EBPβ‐HDO led to a significant enhancement of dopamine concentration in both the striatum and SNc regions of MPTP‐treated A53T mice (Figure 4c,d). Immunofluorescence studies demonstrated that C/EBPβ‐HDO effectively restored diminished TH immunoreactivity in both the striatum and SNc regions of MPTP‐treated A53T mice (Figure 4e,f,j,k). Additionally, using immunofluorescence dual‐labeling assays for TH and C/EBPβ, TH and p‐α‐Syn, TH and α‐Syn, we observed a simultaneous increase in the expression of C/EBPβ, p‐α‐Syn, and α‐Syn, along with a reduction in TH neuron numbers in the SNc area of MPTP‐exposed A53T mice; this phenomenon was alleviated after treatment with C/EBPβ‐HDO (Figure 4g‐i, l‐n). Immunofluorescence analyses of IBA1 and GFAP showed that C/EBPβ‐HDO significantly decreased the elevated immunoreactivity of IBA1 and GFAP in the SNc region of MPTP‐treated A53T mice (Figure S6). Western blot analyses confirmed the increase in TH protein expression and the decrease in C/EBPβ, p‐α‐Syn, and α‐Syn protein expression in the SNc of MPTP‐treated A53T mice following intervention with C/EBPβ‐HDO (Figure 4o). The findings indicate that by directly suppressing the expression of C/EBPβ, C/EBPβ‐HDO therapy can inhibit abnormal α‐Syn production in the SNc area of A53T mice exposed to MPTP. This inhibition ultimately leads to a reduction in the degeneration of dopaminergic neurons.

### 2.5 The activation of Nrf2 through the use of SFN and the suppression of C/EBPβ expression via C/EBPβ‐HDO treatment were found to hinder the aggregation of abnormal α‐Syn in preformed fibrils (PFFs)‐treated HEK293‐α‐Syn‐YFP cells

To investigate the influence of SFN on PFFs‐induced α‐Syn aggregation in HEK293‐α‐Syn‐YFP cells, we activated Nrf2 and evaluated its effects. Our observations revealed the formation of intracellular fluorescence spots in HEK293‐α‐Syn‐YFP cells following exposure to PFFs, indicating the aggregation of α‐Syn. Remarkably, SFN intervention significantly suppressed this aggregative activity (Figure S7A). Moreover, western blot analysis demonstrated that PFFs decreased the levels of Nrf2 expression while increasing those of C/EBPβ, p‐α‐Syn, α‐Syn, insoluble p‐α‐Syn, and insoluble α‐Syn expression. However, when SFN was used as an intervention agent, Nrf2 expression was significantly increased, and the levels of C/EBPβ, p‐α‐Syn, α‐Syn, insoluble p‐α‐Syn, and insoluble α‐Syn were significantly reduced in PFFs‐treated HEK293‐α‐Syn‐YFP cells (Figure S7B). Therefore, our data indicate that SFN‐mediated activation of Nrf2 has the potential to serve as a therapeutic tool against abnormal C/EBPβ expression and α‐Syn aggregation in PFFs‐treated HEK293‐α‐Syn‐YFP cells.

In addition, we conducted experiments to evaluate the impact of C/EBPβ‐HDO administration on α‐Syn aggregation in PFFs‐stimulated HEK293‐α‐Syn‐YFP cells. After treatment with PFFs, YFP‐α‐Syn aggregated into small fluorescent clusters located within intracellular spaces. However, upon therapy with C/EBPβ‐HDO, a corresponding decrease in aggregation levels was observed (Figure S7C). Furthermore, western blotting revealed that C/EBPβ‐HDO administration significantly reduced the increased expression levels of C/EBPβ, p‐α‐Syn, α‐Syn, insoluble p‐α‐Syn, and insoluble α‐Syn in PFFs‐treated HEK293‐α‐Syn‐YFP cells (Figure S7D). Overall, our data demonstrate that the suppression of C/EBPβ expression mediated by C/EBPβ‐HDO leads to a remarkable reduction in abnormal α‐Syn aggregation in PFFs‐exposed HEK293‐α‐Syn‐YFP cells.

### 2.6 C/EBPβ‐HDO‐mediated inhibition of aberrant C/EBPβ expression results in a marked decrease in degeneration of dopaminergic neurons in PFFs‐treated A53T mouse models

**FIGURE 5:**
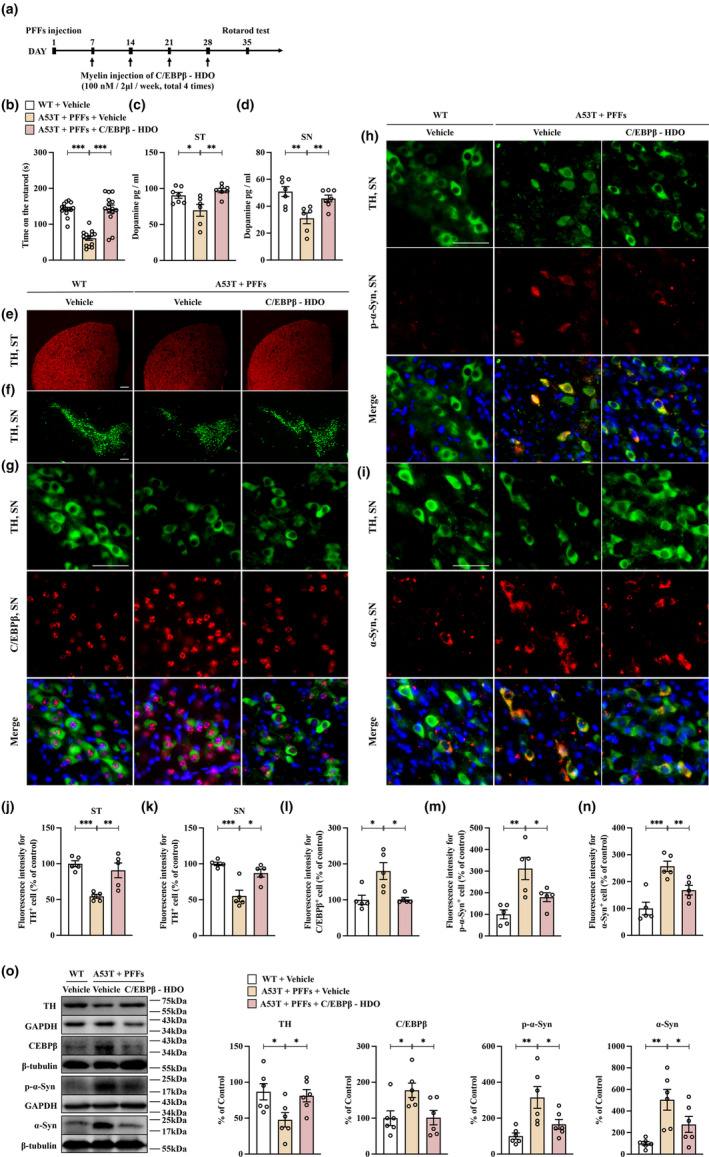
The therapeutic agent C/EBPβ‐HDO can significantly mitigate PD pathology induced by PFFs in vivo. (a) The schedule of treatment. PFFs (3.75 μg/μL, total 5 μg protein) were injected into the right site of substantia nigra. C/EBPβ – HDO (100 nM, 2 μL) was myelin injection on Day 7, Day 14, Day 21, and Day 28. (b) Rotarod test. The duration time for the rotarod test were recorded and compared (mean ± SEM, *n* = 14 per group, one‐way ANOVA, ***p < 0.001). (c,d) C/EBPβ‐HDO attenuated the decreased concentration of dopamine in the striatum and SNc of PFFs‐treated A53T mice. ELISA assay showed that the decreased concentration of dopamine in the striatum and SNc of PFFs‐treated A53T mice were significantly ameliorated by C/EBPβ‐HDO (mean ± SEM, *n* = 6 or 7 per group, one‐way ANOVA, *p < 0.05, **p < 0.01). (e,f) Immunofluorescence staining showed that the TH neuron reduction in both striatum and SNc regions with the treatment of PFFs, which was attenuated by treatment with C/EBPβ‐HDO. Scale bar = 200 μm. (g) Immunofluorescent double staining of TH and C/EBPβ demonstrates that the loss of TH neurons is coupled with an increase of C/EBPβ. Scale bar = 50 μm. (h) Immunofluorescent double staining of TH and p‐α‐Syn demonstrates that the loss of TH neurons is coupled with an increase of p‐α‐Syn. Scale bar = 50 μm. (i) Immunofluorescent double staining of TH and α‐Syn demonstrates that the loss of TH neurons is coupled with an increase of α‐Syn. Scale bar = 50 μm. (j‐n): The immunostaining intensities of TH, C/EBPβ, p‐α‐Syn, and α‐Syn were measured and compared among (mean ± SEM, *n* = 5 per group for TH, C/EBPβ, p‐α‐Syn, and α‐Syn, one‐way ANOVA, *p < 0.05, **p < 0.01, and ***p < 0.001). (o) C/EBPβ‐HDO attenuates the downregulation of TH and Nrf2 and the upregulation of C/EBPβ, p‐α‐Syn, and α‐Syn expression in PFFs‐treated A53T mice. Western blot suggested that administration of C/EBPβ‐HDO significantly ameliorated the downregulation of Nrf2 and TH and the upregulation of C/EBPβ, p‐α‐Syn, and α‐Syn protein expression in the SNc of PFFs‐treated A53T mice (mean ± SEM, *n* = 6 per group, one‐way ANOVA, *p < 0.05 and **p < 0.01).

Injection of human α‐Syn PFFs into the rat SNs, in combination with adeno‐associated virus (AAV)‐mediated overexpression of human α‐Syn, triggers Lewy‐like α‐Syn pathology in DA neurons and impairs motor behavior (Thakur et al., 2017). Conversely, our in vitro findings suggest that targeted suppression of abnormal C/EBPβ expression can hinder the pathological aggregation of α‐Syn in HEK293‐α‐Syn‐YFP cells treated with PFFs. Therefore, this study aimed to investigate the potential of C/EBPβ‐HDO therapy in attenuating the degradation of dopaminergic neurons in the SNc region of A53T mice exposed to PFFs by inhibiting the expression of C/EBPβ. In the PFFs‐treated α‐Syn‐A53T mice, administration of repeated intrathecal injections of C/EBPβ‐HDO at a concentration of 100 nM per 2 μL per week for a total of four times demonstrated a significant enhancement in the duration of the rotarod test (Figure 5a,b). An ELISA study revealed that infusion of C/EBPβ‐HDO led to a significant increase in dopamine levels in both the striatum and SNc regions of PFFs‐treated A53T mice (Figure 5c,d). Additionally, administration of C/EBPβ‐HDO resulted in substantial restoration of TH immunoreactivity within the striatum and SNc regions of PFFs‐exposed mice (Figure 5e,f,j,k). The findings from immunofluorescent co‐staining showed that an increase in C/EBPβ, p‐α‐Syn, and α‐Syn levels accompanied the loss of TH neurons in the SNc region of PFFs‐stimulated A53T mice. However, the impact of these changes was notably lessened with the intervention of C/EBPβ‐HDO (Figure 5g‐i,l‐n). Immunofluorescence results for IBA1 and GFAP indicated that C/EBPβ‐HDO treatment greatly reduced the heightened IBA1 and GFAP immunoreactivity observed in the SNc of PFFs‐exposed A53T mice (Figure S8). Additionally, western blot assays provided further validation for our findings, showing that TH expression levels were upregulated, while C/EBPβ, p‐α‐Syn, α‐Syn, insoluble p‐α‐Syn, and insoluble α‐Syn expression levels were downregulated in the SNc region of PFFs‐treated A53T mice following C/EBPβ‐HDO therapy (Figure 5o and Figure S9). Our findings indicate that the downregulation of C/EBPβ expression can mitigate dopaminergic neuron death in the SNc region of PFFs‐treated A53T mice. This effect is likely attributed to the subsequent inhibition of abnormal α‐Syn expression and its associated pathological effects on cells.

## 3 DISCUSSION

Our study has revealed that Nrf2 inhibits abnormal C/EBPβ transcription in MPP^+^‐treated SH‐SY5Y cells. Administration of SFN, an activator of Nrf2, and silencing of C/EBPβ through C/EBPβ‐HDO significantly reduce levels of abnormal α‐Syn expression in MPP^+^‐stimulated primary neurons. Additionally, administering glucoraphanin to activate Nrf2 results in a significant reduction in abnormal C/EBPβ and α‐Syn expression in the SNc of MPTP‐treated A53T mice, thereby mitigating dopaminergic neuronal degradation. Moreover, our results demonstrate that directly silencing abnormal C/EBPβ expression using C/EBPβ‐HDO can reduce α‐Syn expression levels in the SNc of MPTP‐treated A53T mice, ultimately attenuating dopaminergic neuron degeneration. Importantly, we discovered that inhibiting abnormal α‐Syn expression by suppressing C/EBPβ expression with C/EBPβ‐HDO significantly mitigates dopaminergic neuron degeneration in the SNc region of PFFs‐treated A53T mice. Overall, our findings suggest that targeting the abnormal C/EBPβ/α‐Syn signaling pathway through Nrf2 activation can be an effective therapeutic strategy for alleviating PD‐like pathological features.

Nrf2 acts as a crucial transcriptional mediator for genes related to antioxidant and Phase II detoxification. The administration of SFN, a potent Nrf2 activator, activates the Nrf2 signaling pathway, leading to the production of antioxidant and anti‐inflammatory effects (Cheung & Kong, 2010; Fahey et al., 1997; Lin et al., 2008; Zhang et al., 1994). Studies suggest that Nrf2 activity declines with age, but pharmacological restoration can enhance its transcriptional activity in older subjects. Furthermore, though Nrf2 is typically found in the cytosol, it is also present in the nucleus of age‐matched patients with PD. In the nucleus, Nrf2 helps mitigate oxidative stress by promoting Nrf2‐dependent transcription of antioxidant enzymes in the SNc region of dopaminergic neurons (Jazwa et al., 2011). Therefore, it is evident that Nrf2 has a role in the pathogenesis of PD.

C/EBPβ functions as a critical transcription factor involved in neuroinflammation and has emerged as a potential target for therapeutic interventions in various neurodegenerative diseases (Pulido‐Salgado et al., 2015). Pro‐inflammatory cytokines, such as IL‐1α, IL‐6, and TNF‐α, can activate C/EBPβ, leading to the activation of microglia and exerting detrimental effects on the nervous system (Magalini et al., 1995; Poli, 1998; Pulido‐Salgado et al., 2015; Straccia et al., 2011; Wedel & Ziegler‐Heitbrock, 1995). These findings suggest that suppressing the inflammatory response might confer neuroprotective benefits by attenuating abnormal C/EBPβ expression. Recent studies have identified C/EBPβ as the primary transcription factor responsible for the mRNA and protein expressions of α‐Syn during aging and in PD‐affected brains (Wu et al., 2021). Research conducted on transgenic mice with human wild‐type α‐Syn revealed that overexpression of C/EBPβ led to increased levels of α‐Syn, resulting in augmented PD pathologies and motor disorders. Conversely, depletion of C/EBPβ in similar transgenic mice with human α‐Syn resulted in reduced expression of α‐Syn, leading to mitigated PD pathologies and motor impairments (Wu et al., 2021). Thus, targeting the inhibition of abnormal C/EBPβ transcription may hold potential in preventing PD pathologies. Our research aimed to investigate whether activation of Nrf2 via SFN treatment or Nrf2 overexpression could reduce C/EBPβ transcription in MPP^+^‐treated SH‐SY5Y cells. We used a combination of qPCR, western blotting, luciferase reporter assays, and ChIP analysis to examine the effects. Our findings revealed that both SFN treatment and Nrf2 overexpression attenuated the increased expression of C/EBPβ mRNA and protein, as well as decreased the activation of the C/EBPβ promoter and the dissociation of Nrf2 with the C/EBPβ promoter in MPP^+^‐treated SH‐SY5Y cells. These results provide evidence that activating Nrf2 through SFN treatment or overexpression can inhibit C/EBPβ transcription in MPP^+^‐treated SH‐SY5Y cells. Since pro‐inflammatory cytokines have been shown to promote C/EBPβ expression (Magalini et al., 1995; Poli, 1998; Pulido‐Salgado et al., 2015; Straccia et al., 2011; Wedel & Ziegler‐Heitbrock, 1995), we also examined their levels in MPP^+^‐treated SH‐SY5Y cells and found that both SFN treatment and Nrf2 overexpression attenuated pro‐inflammatory cytokine expression. Therefore, it is possible that Nrf2's ability to bind to the C/EBPβ promoter and inhibit inflammatory reactions contributes to its ability to reduce C/EBPβ transcription in MPP^+^‐treated SH‐SY5Y cells. However, the exact mechanism through which Nrf2 inhibits C/EBPβ transcription remains unclear and warrants further investigation.

Numerous genetic and biochemical studies have demonstrated that α‐Syn is the primary constituent of LBs and plays a central role in the development of PD (Luk et al., 2012; Shahmoradian et al., 2019). Therefore, potential therapeutic approaches for PD may involve strategies aimed at reducing the abnormal expression of α‐Syn. Interestingly, recent investigations have revealed that C/EBPβ acts as a critical transcription factor for α‐Syn in both aging and PD‐affected brains (Wu et al., 2021). Our in vitro study demonstrated that the activation of Nrf2 effectively inhibited the abnormal transcription of C/EBPβ. To further investigate the mechanisms underlying this observation, we treated primary neurons exposed to MPP^+^ with SFN or C/EBPβ‐HDO to activate Nrf2 or silence C/EBPβ. We observed that the activation of Nrf2 via SFN treatment or the silencing of C/EBPβ through C/EBPβ‐HDO attenuated abnormal α‐Syn expression levels in the MPP^+^‐treated primary neurons. Treatment with glucoraphanin, a precursor of SFN, resulted in the downregulation of downstream targets such as C/EBPβ and α‐Syn, consequently promoting extensive survival of dopaminergic neurons. This was accompanied by evident motor improvements in MPTP‐treated A53T mice. Additionally, the activation of Nrf2 through SFN treatment or the silencing of C/EBPβ through C/EBPβ‐HDO efficiently suppressed Lewy‐like pathology in HEK293‐α‐Syn‐YFP cells treated with PFFs. Silencing the abnormal expression of C/EBPβ through the inhibition of abnormal α‐Syn also facilitated the survival of dopaminergic neurons and ameliorated motor deficits in A53T mice treated with PFFs. Our findings suggest that the activation of Nrf2 or the silencing of abnormal C/EBPβ expression can lead to reduced aberrant α‐Syn levels, thereby alleviating α‐Syn‐induced pathological changes.

Chronic neuroinflammation is a crucial pathogenic factor implicated in various neurodegenerative disorders. It primarily acts by increasing the levels of glia‐derived cytokines, which trigger neurotoxic effects on vulnerable dopaminergic neurons (Barcia et al., 2003; Halliday & Stevens, 2011; Taylor et al., 2013). Reactive microglia/astrocytes expressing IBA1/GFAP have been observed in the SNc region of both mouse models and patients with PD, suggesting a potential involvement of gliosis‐derived inflammatory processes in the development of PD pathology (Costa et al., 2013). Consistent with this observation, inhibiting the glial activation‐mediated inflammatory response can provide protection to dopaminergic neurons both in vivo and in vitro (Wi et al., 2020). In PD mouse models, oral administration of 0.1% glucoraphanin and repeated intrathecal injection of C/EBPβ‐HDO successfully prevented glial activation and reduced degeneration of TH neurons in the SNc. These findings strongly suggest that the neuroprotective effects of glucoraphanin and C/EBPβ‐HDO may arise from their ability to inhibit glial activation in the SNc region of PD mouse models.

In conclusion, our data support the effectiveness of Nrf2 activation in suppressing abnormal C/EBPβ transcription. The activation of Nrf2 through its activator or the inhibition of C/EBPβ using C/EBPβ‐HDO can effectively reduce the degeneration of dopaminergic neurons in mouse models of PD by decreasing abnormal α‐Syn expression levels. Based on these findings, it can be inferred that blocking the aberrant C/EBPβ/α‐Syn signaling pathway through Nrf2 activation could serve as a promising therapeutic strategy for PD. Thus, Nrf2 activators like SFN and glucoraphanin, along with C/EBPβ‐HDO, show great potential as therapeutic agents for combating PD.

## 4 MATERIALS AND METHODS

### 4.1 Mice and cell lines

Male transgenic mice expressing A53T human α‐Syn and wild type mice (12 weeks old, 25–30 g) were used for the experiments. The animals were housed under controlled temperature and kept in a 12‐h light/dark cycle with ad libitum access to food and water. The animal protocol was approved by the Jinan University Institutional Animal Care and Use Committee, and all experiments were performed following the Guide for Animal Experimentation of Jinan University.

Primary rat neurons were cultured as previously described (Kim et al., 2017; Wu et al., 2021). All rats were purchased from the Guangdong Experimental Animal Center, China. The protocol was reviewed and approved by the Jinan University Institutional Animal Care and Use Committee. During embryonic Day 15, we dissected the brains of fetal rats and exposed it to a cold Hank's equilibrium salt solution that was free of Ca^2+^/Mg^2+^ (HBSS). We used 4 mL of HBSS per embryo. The tissue was dissociated using 0.05% trypsin in HBSS for 10 min at 37°C and subsequently centrifuged at 300× *g* for 2 min. The tissue was re‐suspended in minimal essential medium containing 10% fetal bovine serum, 10% heat‐inactivated horse serum, 2 mM L‐glutamine, and 1% penicillin/streptomycin. Lastly, it was filtered through a 70 μm cell strainer twice. Around 5 × 10^7^ neurons were extracted from 20 to 30 embryos. The neurons were cultured on poly‐D‐lysine‐coated plates at a concentration of 0.05 mg/mL and incubated at 37°C with serum‐free neurobasal media containing 2% B27 supplement, 1% L‐glutamine and 1% penicillin/streptomycin for using.

SH‐SY5Y cells and HEK293T cells stably expressing YFP‐labeled human α‐synuclein (HEK293‐α‐Syn‐YFP) were cultured in DMEM/F‐12 (basal media) or DMEM supplemented with 10% fetal bovine serum (Excell Bio.) and 1%penicillin/streptomycin. Cells were cultured at 37°C in a humidified incubator containing 5% CO_2_.

### 4.2 Materials

MPP^+^ (1‐methyl‐4‐phenylpyridinium), MPTP (1‐methyl‐4‐phenyl‐1,2,5,6‐tetrahydropyridine), and glucoraphanin was purchased from MedChemExpress. MPP^+^ and MPTP was dissolved in phosphate‐buffered saline (PBS) or 0.9% sterile saline. Sulforaphane (SFN, LKT Laboratories, Inc.) was dissolved in distilled water containing 10% corn oil; glucoraphanin (MedChemExpress) was dissolved in H_2_O. SFN (1 μM) and/or MPP^+^ (1 mM) was treated for in vitro study. MPTP (30 mg/kg) was administered intraperitoneally (i.p.) to mice. 0.1% glucoraphanin was administered oral to mice. The doses of MPTP (30 mg/kg), MPP^+^ (1 mM), SFN (1 μM), and 0.1% glucoraphanin selected correspond to those previously reported (Kang et al., 2018; Yao et al., 2016). The mouse pcDNA3.1‐*Nrf2* plasmids were kindly gifted by Dr. Masayuki Yamamoto (Tohoku University Graduate School of Medicine, Sendai, Japan).

Antisense oligonucleotides (ASOs) for C/EBPβ and cRNA were purchased from TsingKe Biological Technology and solubilized in 0.9% sterile saline before use. For the production of C/EBPβ‐DNA/RNA heteroduplex oligonucleotide (HDO), equimolar amounts of DNA and cRNA strands were heated in 0.9% sterile saline at 95°C for 5 min and slowly cooled to room temperature. C/EBPβ‐HDO harbored locked nucleic acids (LNAs) at each end flanking the central base of DNA with or without a CY5 label, and C/EBPβ‐HDO harbored 2′‐O‐methyl at each end flanking the central base of cRNA. The sequences of ASOs and cRNA targeting α‐Syn used in our experiments are as follows: ASO‐C/EBPβ, C(L)^C(L)^A^G^C^A^G^G^C^G^G^T^G^C^A^T^G^A(L)^A(L)^C(L) (Tronel et al., 2005); cRNA, G(M)^U(M)^U(M)^CAUGCACCGCCUGCUG(M)^G(M), where L indicates the locked nucleic acids; M indicates the 2′‐O‐methyl modifications; and ^ indicates the phosphorothioate bond. SH‐SY5Y cells or HEK293‐α‐Syn‐YFP cells were transfected with different doses of C/EBPβ‐HDO for 24 h using Lipofectamine 3000 (Invitrogen) according to the manufacturer's instructions. After transfection for 24 h, cells were collected for qPCR, western blot assays, and immunofluorescence staining.

Full‐length human α‐Syn was expressed in BL21 (DE3) competent *Escherichia coli* (Life Technologies) and purified as previously described (Volpicelli‐Daley et al., 2014). The purified recombinant α‐Syn was stored at −80°C until use. Preformed fibrils (PFFs) were made by diluting recombinant α‐Syn to 5 mg/mL in sterile Dulbecco's PBS (Cellgro, Mediatech; pH adjusted to 7.0, without Ca^2+^ or Mg^2+^) followed by incubation at 37°C with constant agitation at 1000 rpm for 7 days. PFFs were sonicated with a water‐bath cup‐horn sonicator (Fisher Scientific) at 50% power for 5 min before use. PFFs (3.75 μg/μL, each 6‐well plate is 2 μL) were transduced using Lipofectamine 3000 (Invitrogen) according to the manufacturer's instructions. After transfection for 24 h, cells were collected for western blot assays and immunofluorescence staining.

### 4.3 Treatment with PFFs and C/EBPβ‐HDO in vivo

Mice were anesthetized with isoflurane and fixed to a stereotaxic apparatus. PFFs (3.75 μg/μL, total 5 μg protein) or sterile PBS were injected into the right site of substantia nigra (1.2 mm lateral, −4.3 mm ventral, and −3.1 mm from Bregma; Kang, Zhang, Liu, Manfredsson, He, et al., 2017) by using a 10 μL Hamilton syringe with a fixed needle at a rate of 0.25 μL/min using a microinjector pump (KDS, Stoelting). The needle remained in place for 5 min after the PFFs were completely injected followed by slow removal (over 2 min). The mice were placed on a heating pad until recovery from anesthesia.

The intrathecal administration of C/EBPβ‐HDO was conducted following the previously described method (Ferreira et al., 1999). The mice were anesthetized with isoflurane, and they received an injection of C/EBPβ‐HDO (100 nM/2 μL) or 0.9% sterile saline solution between the L5 and L6 vertebrae using a 10 μL Hamilton syringe. Subsequently, the animals were placed on a heating pad, providing thermal support until full recovery from anesthesia.

### 4.4 Luciferase reporter assay

SH‐SY5Y cells were cotransfected with C/EBP β promoter luciferase reporter plasmid together with pRL‐TK Renilla luciferase plasmid (Promega), MPP^+^, SFN, and/or pcDNA3‐*Nrf2* plasmid. After administration for 24 h, cells were collected and analyzed using the dual‐luciferase reporter assay kit (Promega) according to the manufacturer's protocol.

### 4.5 Chromatin immunoprecipitation (ChIP) assay

The ChIP assay was performed as described previously (Wang et al., 2018; Wu et al., 2020). Cells samples were subjected to ChIP assay according to the manual of the SimpleChIP® Enzymatic Chromatin IP Kit (Cell Signaling). Specifically, 7.5 μg of Nrf2 antibody (Abcam) was added to the homogenate of the cell lysates. The mixture was incubated overnight at 4°C. The washing, elution, and reverse cross‐linking of free DNA were performed according to the manufacturer's protocol. *C/EBPβ* promoter‐specific primers were used to amplify the promoter region. The primer sequences were as follows: forward 5′ GGCAGGGGGCGTGAGG 3′; reverse 5′ GGTCCCCTTCCCAGTCCC′. The PCR amplicons were separated on a 2% agarose gel after 35 cycles of PCR (denaturation at 95°C for 30 s, annealing at 58°C for 30 s, and extension at 72°C for 30 s).

### 4.6 Quantitative PCR (qPCR) assay

Levels of *C/EBPβ* mRNA were examined by qPCR. RNA was extracted using an Eastep® Super Kit (Promega) followed by reverse transcription with GoScript™ Reverse Transcriptase Mix, Oligo (dT; Promega) to generate cDNA. The qPCR assays were performed with the ChamQ™ SYBR® qPCR Master Mix Kit (Vazyme) using the 788BR05175 Real‐Time PCR System. The PCR amplification protocol was as follows: 40 cycles of denaturation at 95°C for 30 s, annealing at 55°C for 30 s, and extension for 30 s at 72°C. The target genes were analyzed by the 2^−ΔΔCt^ method. The primer sequences were as follows: human *C/EBPβ* forward, 5′ GACAAGCACAGCGACGAGTA 3′; human *C/EBPβ* reverse, 5′ AGCTGCTCCACCTTCTTCTG 3′; mouse *C/EBPβ* forward, 5′ CAACCTGGAGACGCAGCACAAG 3′; mouse *C/EBPβ* reverse, 5′ GCTTGAACAAGTTCCGCAGGGT 3′.

### 4.7 Western blot analysis

Cells and brain homogenates were lysed in RIPA buffer. The samples from the nucleus and cytoplasm were extracted using a kit (P0027, Beyotime). Protein concentrations were determined by a Coomassie Brilliant Blue protein assay kit (Bio–Rad). Total protein (10 μg) was separated on 10%–12% sodium dodecyl sulfate‐polyacrylamide gels and then transferred to polyvinylidene difluoride (PVDF) membranes. The membranes were blocked with 5% milk at room temperature for 1 h followed by incubation with primary antibodies at 4°C for 12 h. Membranes were then washed three times with TBST and incubated with the corresponding secondary antibody for 1 h at room temperature. After an additional three washes, targeted proteins were detected using the enhanced chemiluminescence method scanned by the Tanon‐5200 CE imaging system (Tanon). The expression levels of target proteins were normalized to β‐actin, β‐tubulin or GAPDH as a loading control. The following primary antibodies were used: anti‐Nrf2 antibody (1:1000, ab137550), anti‐C/EBP β antibody (1:1000, ab32358), anti‐p‐α‐Syn (1:1000, ab51253), anti‐α‐Syn antibody (1:1000, ab1903), and anti‐Lamin B antibody (1:1000, ab16048) were purchased from Abcam; anti‐tyrosine‐hydroxylase (TH) antibody (1:1000, GTX10372) was purchased from GTX (GeneTex); anti‐β‐actin antibody (ab198991) and anti‐β‐tubulin antibody (ab176560) were purchased from Abcam; anti‐GAPDH antibody (AF7021) was purchased from Affinity. The HRP‐conjugated anti‐rabbit/mouse IgG antibody was purchased from Abbkine.

### 4.8 Sequential extraction

The soluble and insoluble fractions of samples were prepared based on the method described earlier (Zhou et al., 2023). Briefly, the cells and brain samples were scraped into a solution containing 1% Triton X‐100 (TX‐100) in Tris‐buffered saline (TBS) (50 mM Tris, 150 mM NaCl, pH 7.4), along with protease and phosphatase inhibitor cocktail; followed by an incubation period of 30 min on ice. The lysates were subjected to sonication and centrifugation at 100,000 *g* for 30 min. The obtained supernatant was regarded as the soluble fraction. Subsequently, the pellet was washed with 1% Triton X‐100 (TX‐100) in Tris‐buffered saline (TBS), sonicated, and centrifuged at 100,000 *g* for 30 min to remove unwanted materials. Next, the supernatant was discarded, and the remaining pellet was considered as the insoluble fraction after being dissolved in 2% SDS in TBS solution.

### 4.9 Enzyme‐linked immunosorbent assay (ELISA)

Cells were homogenized and lysed using RIPA buffer followed by centrifugation at 15,000 × g for 4 min at 4°C. The resulting supernatant was used to perform an ELISA assay after dilution of samples in a 10‐fold concentration of an ELISA diluent solution. Levels of interleukin‐1β (IL‐1β) and interleukin‐6 (IL‐6) were then quantified utilizing an ELISA kit (Excell Bio), following the manufacturer's instructions. Brain samples were homogenized and lysed in phosphate‐buffered saline (PBS), followed by centrifugation at 5000 × g for 5 min at 4°C. The resulting supernatant was then used to perform an ELISA assay. Dopamine levels were quantified using an ELISA kit (Elabscience), following the manufacturer's instructions.

### 4.10 Immunofluorescence staining

The cells or brain sections from mice were pre‐planted on glass slides and fixed using 4% paraformaldehyde (PFA) at room temperature for 10 min. The slides were washed with PBS 3 times, followed by blocking using 3% BSA with 0.3% Triton X‐100 for half an hour. Then, the samples were incubated with primary antibodies against TH (GTX10372 and AF6113, 1:500), C/EBPβ (ab32358, 1:500), p‐α‐Syn (ab51253, 1:500), α‐Syn (ab1903, 1:500), IBA1 (ab283319, 1:500), and GFAP (Affinity, DF6040, 1:500) for 48 h at 4°C. The cells and brain sections were then washed with PBS and incubated with Alexa Fluor 488/594 anti‐mouse/rabbit secondary antibody (1:500) for 2 h at room temperature in the dark, followed by staining with DAPI to visualize the nuclei. Visualization of cells and brain sections were done using a fluorescence microscope (Olympus BX53).

### 4.11 Rotarod test

For the rotarod test, mice were trained for three sequential days on the rotarod. Each daily practice session consisted of placing the subject on the rotarod at a slow rotational speed (5 rpm) for a maximum of 5 min. Mice were given three test trials on the test day. The rotational speed of rotarod was modulated from 0 rpm to a maximum 40 rpm. It was gradually increased during the trial at a rate of 0.1 rpm/s. Each trial was started and then sustained for 5 min. The trial was stopped when the mouse fell (activating a switch that automatically stopped the timer) or when 5 min had elapsed. The residence time on the rotarod was counted using a stopwatch. The results showed the average value of the three trials.

### 4.12 Statistical analysis

All data results are expressed as the mean ± standard error of the mean (SEM) and were analyzed using PASW Statistics 20 software (formerly SPSS Statistics, SPSS). Potential differences between the mean values were evaluated using one‐way analysis of variance followed by post hoc Fisher's least significant difference test. Student's *t*‐test was used to compare the differences between two groups unless otherwise specified. Asterisks were used to indicate significance: *p < 0.05, **p < 0.01, and ***p < 0.001. Values >0.05 were considered not significant (ns).

## AUTHOR CONTRIBUTIONS

JZ and WY conceived of the project, designed the experiments, and wrote the manuscript. ZL performed most of the experiments and analyzed the data. LH, QC, and HL assisted with data analysis and interpretation and critically read the manuscript. All authors read and approved the final manuscript.

## FUNDING INFORMATION

This work is supported by the National Natural Science Foundation of China (82271563 to JC, 82204786 to WY), the Natural Science Foundation of Guangdong Province of China (2022A1515012512 to JZ), and the Academic Promotion Program of Shandong First Medical University.

## CONFLICT OF INTEREST STATEMENT

The authors declare that they have no conflicts of interest.

## Supporting information


Figures S1‐S9

## Data Availability

The datasets used and/or analyzed during the current study are available from the corresponding author on reasonable request.

## References

[acel13958-bib-0001] Asada, K. , Sakaue, F. , Nagata, T. , Zhang, J. C. , Yoshida‐Tanaka, K. , Abe, A. , Nawa, M. , Nishina, K. , & Yokota, T. (2021). Short DNA/RNA heteroduplex oligonucleotide interacting proteins are key regulators of target gene silencing. Nucleic Acids Research, 49(9), 4864–4876. 10.1093/nar/gkab258 33928345 PMC8136785

[acel13958-bib-0002] Barcia, C. , Fernandez Barreiro, A. , Poza, M. , & Herrero, M. T. (2003). Parkinson's disease and inflammatory changes. Neurotoxicity Research, 5(6), 411–418. 10.1007/BF03033170 14715444

[acel13958-bib-0003] Cheung, K. L. , & Kong, A. N. (2010). Molecular targets of dietary phenethyl isothiocyanate and sulforaphane for cancer chemoprevention. The AAPS Journal, 12(1), 87–97. 10.1208/s12248-009-9162-8 20013083 PMC2811646

[acel13958-bib-0004] Costa, G. , Frau, L. , Wardas, J. , Pinna, A. , Plumitallo, A. , & Morelli, M. (2013). MPTP‐induced dopamine neuron degeneration and glia activation is potentiated in MDMA‐pretreated mice. Movement Disorders, 28(14), 1957–1965. 10.1002/mds.25646 24108425

[acel13958-bib-0005] Dauer, W. , & Przedborski, S. (2003). Parkinson's disease: Mechanisms and models. Neuron, 39(6), 889–909. 10.1016/s0896-6273(03)00568-3 12971891

[acel13958-bib-0006] Ellis, C. E. , Schwartzberg, P. L. , Grider, T. L. , Fink, D. W. , & Nussbaum, R. L. (2001). Alpha‐Synuclein is phosphorylated by members of the Src family of protein‐tyrosine kinases. Journal of Biological Chemistry, 276(6), 3879–3884. 10.1074/jbc.M010316200 11078745

[acel13958-bib-0007] Fahey, J. W. , Zhang, Y. , & Talalay, P. (1997). Broccoli sprouts: An exceptionally rich source of inducers of enzymes that protect against chemical carcinogens. Proceedings of the National Academy of Sciences of the United States of America, 94(19), 10367–10372. 10.1073/pnas.94.19.10367 9294217 PMC23369

[acel13958-bib-0008] Ferreira, J. , Santos, A. R. , & Calixto, J. B. (1999). The role of systemic, spinal and supraspinal L‐arginine‐nitric oxide‐cGMP pathway in thermal hyperalgesia caused by intrathecal injection of glutamate in mice. Neuropharmacology, 38(6), 835–842. 10.1016/s0028-3908(99)00006-4 10465687

[acel13958-bib-0009] Gomez‐Santos, C. , Barrachina, M. , Gimenez‐Xavier, P. , Dalfo, E. , Ferrer, I. , & Ambrosio, S. (2005). Induction of C/EBP beta and GADD153 expression by dopamine in human neuroblastoma cells. Relationship with alpha‐synuclein increase and cell damage. Brain Research Bulletin, 65(1), 87–95. 10.1016/j.brainresbull.2004.11.008 15680548

[acel13958-bib-0010] Halliday, G. M. , & Stevens, C. H. (2011). Glia: Initiators and progressors of pathology in Parkinson's disease. Movement Disorders, 26(1), 6–17. 10.1002/mds.23455 21322014

[acel13958-bib-0011] Jazwa, A. , Rojo, A. I. , Innamorato, N. G. , Hesse, M. , Fernandez‐Ruiz, J. , & Cuadrado, A. (2011). Pharmacological targeting of the transcription factor Nrf2 at the basal ganglia provides disease modifying therapy for experimental parkinsonism. Antioxidants & Redox Signaling, 14(12), 2347–2360. 10.1089/ars.2010.3731 21254817

[acel13958-bib-0012] Jiang, P. , Gan, M. , Ebrahim, A. S. , Castanedes‐Casey, M. , Dickson, D. W. , & Yen, S. H. (2013). Adenosine monophosphate‐activated protein kinase overactivation leads to accumulation of alpha‐synuclein oligomers and decrease of neurites. Neurobiology of Aging, 34(5), 1504–1515. 10.1016/j.neurobiolaging.2012.11.001 23200460 PMC3570625

[acel13958-bib-0013] Kang, S. S. , Ahn, E. H. , Zhang, Z. T. , Liu, X. , Manfredsson, F. P. , Sandoval, I. M. , Dhakal, S. , Iuvone, P. M. , Cao, X. , & Ye, K. Q. (2018). Alpha‐Synuclein stimulation of monoamine oxidase‐B and legumain protease mediates the pathology of Parkinson's disease. EMBO Journal, 37(12), e98878. 10.15252/embj.201798878 29769405 PMC6003650

[acel13958-bib-0014] Kang, S. S. , Zhang, Z. , Liu, X. , Manfredsson, F. P. , Benskey, M. J. , Cao, X. , Xu, J. , Sun, Y. E. , & Ye, K. (2017). TrkB neurotrophic activities are blocked by alpha‐synuclein, triggering dopaminergic cell death in Parkinson's disease. Proceedings of the National Academy of Sciences of the United States of America, 114(40), 10773–10778. 10.1073/pnas.1713969114 28923922 PMC5635931

[acel13958-bib-0015] Kang, S. S. , Zhang, Z. , Liu, X. , Manfredsson, F. P. , He, L. , Iuvone, P. M. , Cao, X. , Sun, Y. E. , Jin, L. , & Ye, K. (2017). Alpha‐Synuclein binds and sequesters PIKE‐L into Lewy bodies, triggering dopaminergic cell death via AMPK hyperactivation. Proceedings of the National Academy of Sciences of the United States of America, 114(5), 1183–1188. 10.1073/pnas.1618627114 28096359 PMC5293033

[acel13958-bib-0016] Kim, J. , Lee, S. , Choi, B. R. , Yang, H. , Hwang, Y. , Park, J. H. , LaFerla, F. M. , Han, J. S. , Lee, K. W. , & Kim, J. (2017). Sulforaphane epigenetically enhances neuronal BDNF expression and TrkB signaling pathways. Molecular Nutrition & Food Research, 61(2). 10.1002/mnfr.201600194 27735126

[acel13958-bib-0017] Kobayashi, E. , Suzuki, T. , & Yamamoto, M. (2013). Roles nrf2 plays in myeloid cells and related disorders. Oxidative Medicine and Cellular Longevity, 2013, 529219. 10.1155/2013/529219 23819012 PMC3684031

[acel13958-bib-0018] Kometsi, L. , Govender, K. , Mofo Mato, E. P. , Hurchund, R. , & Owira, P. M. O. (2020). By reducing oxidative stress, naringenin mitigates hyperglycaemia‐induced upregulation of hepatic nuclear factor erythroid 2‐related factor 2 protein. The Journal of Pharmacy and Pharmacology, 72, 1394–1404. 10.1111/jphp.13319 32628779

[acel13958-bib-0019] Lashuel, H. A. , Overk, C. R. , Oueslati, A. , & Masliah, E. (2013). The many faces of α‐synuclein: From structure and toxicity to therapeutic target. Nature Reviews. Neuroscience, 14(1), 38–48. 10.1038/nrn3406 23254192 PMC4295774

[acel13958-bib-0020] Lin, W. , Wu, R. T. , Wu, T. , Khor, T. O. , Wang, H. , & Kong, A. N. (2008). Sulforaphane suppressed LPS‐induced inflammation in mouse peritoneal macrophages through Nrf2 dependent pathway. Biochemical Pharmacology, 76(8), 967–973. 10.1016/j.bcp.2008.07.036 18755157 PMC2577694

[acel13958-bib-0021] Luk, K. C. , Kehm, V. , Carroll, J. , Zhang, B. , O'Brien, P. , Trojanowski, J. Q. , & Lee, V. M. (2012). Pathological alpha‐synuclein transmission initiates Parkinson‐like neurodegeneration in nontransgenic mice. Science, 338(6109), 949–953. 10.1126/science.1227157 23161999 PMC3552321

[acel13958-bib-0022] Luo, S. , Kang, S. S. , Wang, Z. H. , Liu, X. , Day, J. X. , Wu, Z. , Peng, J. , Xiang, D. , Springer, W. , & Ye, K. (2019). Akt phosphorylates NQO1 and triggers its degradation, abolishing its antioxidative activities in Parkinson's disease. The Journal of Neuroscience, 39(37), 7291–7305. 10.1523/JNEUROSCI.0625-19.2019 31358653 PMC6759025

[acel13958-bib-0023] Ma, Q. (2013). Role of nrf2 in oxidative stress and toxicity. Annual Review of Pharmacology and Toxicology, 53, 401–426. 10.1146/annurev-pharmtox-011112-140320 PMC4680839 23294312

[acel13958-bib-0024] Magalini, A. , Savoldi, G. , Ferrari, F. , Garnier, M. , Ghezzi, P. , Albertini, A. , & Di Lorenzo, D. (1995). Role of IL‐1 beta and corticosteroids in the regulation of the C/EBP‐alpha, beta and delta genes in vivo. Cytokine, 7(8), 753–758. 10.1006/cyto.1995.0090 8664441

[acel13958-bib-0025] Nishina, K. , Piao, W. , Yoshida‐Tanaka, K. , Sujino, Y. , Nishina, T. , Yamamoto, T. , Nitta, K. , Yoshioka, K. , Kuwahara, H. , Yasuhara, H. , & Yokota, T. (2015). DNA/RNA heteroduplex oligonucleotide for highly efficient gene silencing. Nature Communications, 6, 7969. 10.1038/ncomms8969 PMC4918363 26258894

[acel13958-bib-0026] Okochi, M. , Walter, J. , Koyama, A. , Nakajo, S. , Baba, M. , Iwatsubo, T. , Meijer, L. , Kahle, P. J. , & Haass, C. (2000). Constitutive phosphorylation of the Parkinson's disease associated alpha‐synuclein. Journal of Biological Chemistry, 275(1), 390–397. 10.1074/jbc.275.1.390 10617630

[acel13958-bib-0027] Poli, V. (1998). The role of C/EBP isoforms in the control of inflammatory and native immunity functions. Journal of Biological Chemistry, 273(45), 29279–29282. 10.1074/jbc.273.45.29279 9792624

[acel13958-bib-0028] Pulido‐Salgado, M. , Vidal‐Taboada, J. M. , & Saura, J. (2015). C/EBPbeta and C/EBPdelta transcription factors: Basic biology and roles in the CNS. Progress in Neurobiology, 132, 1–33. 10.1016/j.pneurobio.2015.06.003 26143335

[acel13958-bib-0029] Shahmoradian, S. H. , Lewis, A. J. , Genoud, C. , Hench, J. , Moors, T. E. , Navarro, P. P. , Castaño‐Díez, D. , Schweighauser, G. , Graff‐Meyer, A. , Goldie, K. N. , Sütterlin, R. , Huisman, E. , Ingrassia, A. , Gier, Y. , Rozemuller, A. J. M. , Wang, J. , Paepe, A. , Erny, J. , Staempfli, A. , … Lauer, M. E. (2019). Lewy pathology in Parkinson's disease consists of crowded organelles and lipid membranes. Nature Neuroscience, 22(7), 1099–1109. 10.1038/s41593-019-0423-2 31235907

[acel13958-bib-0030] Straccia, M. , Gresa‐Arribas, N. , Dentesano, G. , Ejarque‐Ortiz, A. , Tusell, J. M. , Serratosa, J. , Solà, C. , & Saura, J. (2011). Pro‐inflammatory gene expression and neurotoxic effects of activated microglia are attenuated by absence of CCAAT/enhancer binding protein beta. Journal of Neuroinflammation, 8, 156. 10.1186/1742-2094-8-156 22074460 PMC3223504

[acel13958-bib-0031] Suzuki, T. , Motohashi, H. , & Yamamoto, M. (2013). Toward clinical application of the Keap1‐Nrf2 pathway. Trends in Pharmacological Sciences, 34(6), 340–346. 10.1016/j.tips.2013.04.005 23664668

[acel13958-bib-0032] Suzuki, T. , & Yamamoto, M. (2015). Molecular basis of the Keap1‐Nrf2 system. Free Radical Biology & Medicine, 88(Pt B), 93–100. 10.1016/j.freeradbiomed.2015.06.006 26117331

[acel13958-bib-0033] Taylor, J. M. , Main, B. S. , & Crack, P. J. (2013). Neuroinflammation and oxidative stress: co‐conspirators in the pathology of Parkinson's disease. Neurochemistry International, 62(5), 803–819. 10.1016/j.neuint.2012.12.016 23291248

[acel13958-bib-0034] Thakur, P. , Breger, L. S. , Lundblad, M. , Wan, O. W. , Mattsson, B. , Luk, K. C. , Lee, V. M. Y. , Trojanowski, J. Q. , & Bjorklund, A. (2017). Modeling Parkinson's disease pathology by combination of fibril seeds and alpha‐synuclein overexpression in the rat brain. Proceedings of the National Academy of Sciences of the United States of America, 114(39), E8284–E8293. 10.1073/pnas.1710442114 28900002 PMC5625925

[acel13958-bib-0035] Toffoli, M. , Vieira, S. R. L. , & Schapira, A. H. V. (2020). Genetic causes of PD: A pathway to disease modification. Neuropharmacology, 170, 108022. 10.1016/j.neuropharm.2020.108022 32119885

[acel13958-bib-0036] Tronel, S. , Milekic, M. H. , & Alberini, C. M. (2005). Linking new information to a reactivated memory requires consolidation and not reconsolidation mechanisms. PLoS Biology, 3(9), e293. 10.1371/journal.pbio.0030293 16104829 PMC1188238

[acel13958-bib-0037] Volpicelli‐Daley, L. A. , Luk, K. C. , & Lee, V. M. (2014). Addition of exogenous α‐synuclein preformed fibrils to primary neuronal cultures to seed recruitment of endogenous α‐synuclein to Lewy body and Lewy neurite‐like aggregates. Nature Protocols, 9(9), 2135–2146. 10.1038/nprot.2014.143 25122523 PMC4372899

[acel13958-bib-0038] Volpicelli‐Daley, L. A. , Luk, K. C. , Patel, T. P. , Tanik, S. A. , Riddle, D. M. , Stieber, A. , Meaney, D. F. , Trojanowski, J. Q. , & Lee, V. M. (2011). Exogenous α‐synuclein fibrils induce Lewy body pathology leading to synaptic dysfunction and neuron death. Neuron, 72(1), 57–71. 10.1016/j.neuron.2011.08.033 21982369 PMC3204802

[acel13958-bib-0039] Wang, Z. H. , Gong, K. , Liu, X. , Zhang, Z. , Sun, X. , Wei, Z. Z. , Yu, S. P. , Manfredsson, F. P. , Sandoval, I. M. , Johnson, P. F. , Jia, J. , Wang, J. Z. , & Ye, K. (2018). C/EBPbeta regulates delta‐secretase expression and mediates pathogenesis in mouse models of Alzheimer's disease. Nature Communications, 9(1), 1784. 10.1038/s41467-018-04120-z PMC5934399 29725016

[acel13958-bib-0040] Wedel, A. , & Ziegler‐Heitbrock, H. W. (1995). The C/EBP family of transcription factors. Immunobiology, 193(2–4), 171–185. 10.1016/s0171-2985(11)80541-3 8530141

[acel13958-bib-0041] Wi, R. , Chung, Y. C. , & Jin, B. K. (2020). Functional crosstalk between CB and TRPV1 receptors protects nigrostriatal dopaminergic neurons in the MPTP model of Parkinson's disease. Journal of Immunology Research, 2020, 5093493. 10.1155/2020/5093493 33062722 PMC7539109

[acel13958-bib-0042] Wu, Z. , Xia, Y. , Wang, Z. , Su Kang, S. , Lei, K. , Liu, X. , Jin, L. , Wang, X. , Cheng, L. , & Ye, K. (2020). C/EBPbeta/delta‐secretase signaling mediates Parkinson's disease pathogenesis via regulating transcription and proteolytic cleavage of alpha‐synuclein and MAOB. Molecular Psychiatry, 26, 568–585. 10.1038/s41380-020-0687-7 32086435

[acel13958-bib-0043] Wu, Z. , Xia, Y. , Wang, Z. , Su Kang, S. , Lei, K. , Liu, X. , Jin, L. , Wang, X. , Cheng, L. , & Ye, K. (2021). C/EBPbeta/delta‐secretase signaling mediates Parkinson's disease pathogenesis via regulating transcription and proteolytic cleavage of alpha‐synuclein and MAOB. Molecular Psychiatry, 26(2), 568–585. 10.1038/s41380-020-0687-7 32086435

[acel13958-bib-0044] Yamamoto, M. , Kensler, T. W. , & Motohashi, H. (2018). The KEAP1‐NRF2 system: A thiol‐based sensor‐effector apparatus for maintaining redox homeostasis. Physiological Reviews, 98(3), 1169–1203. 10.1152/physrev.00023.2017 29717933 PMC9762786

[acel13958-bib-0045] Yang, J. , Luo, S. , Zhang, J. , Yu, T. , Fu, Z. , Zheng, Y. , Xu, X. , Liu, C. , Fan, M. , & Zhang, Z. (2020). Exosome‐mediated delivery of antisense oligonucleotides targeting alpha‐synuclein ameliorates the pathology in a mouse model of Parkinson's disease. Neurobiology of Disease, 148, 105218. 10.1016/j.nbd.2020.105218 33296726

[acel13958-bib-0046] Yao, W. , Zhang, J. C. , Ishima, T. , Dong, C. , Yang, C. , Ren, Q. , Ma, M. , Han, M. , Wu, J. , Suganuma, H. , Ushida, Y. , Yamamoto, M. , & Hashimoto, K. (2016). Role of Keap1‐Nrf2 signaling in depression and dietary intake of glucoraphanin confers stress resilience in mice. Scientific Reports, 6, 30659. 10.1038/srep30659 27470577 PMC4965765

[acel13958-bib-0047] Yoshioka, K. , Kunieda, T. , Asami, Y. , Guo, H. , Miyata, H. , Yoshida‐Tanaka, K. , Sujino, Y. , Piao, W. , Kuwahara, H. , Nishina, K. , Hara, R. I. , Nagata, T. , Wada, T. , Obika, S. , & Yokota, T. (2019). Highly efficient silencing of microRNA by heteroduplex oligonucleotides. Nucleic Acids Research, 47(14), 7321–7332. 10.1093/nar/gkz492 31214713 PMC6698647

[acel13958-bib-0048] Zhang, J. , Li, X. , & Li, J. D. (2019). The roles of post‐translational modifications on α‐Synuclein in the pathogenesis of Parkinson's diseases. Frontiers in Neuroscience, 13, 381. 10.3389/fnins.2019.00381 31057362 PMC6482271

[acel13958-bib-0049] Zhang, Y. , Kensler, T. W. , Cho, C. G. , Posner, G. H. , & Talalay, P. (1994). Anticarcinogenic activities of sulforaphane and structurally related synthetic norbornyl isothiocyanates. Proceedings of the National Academy of Sciences of the United States of America, 91(8), 3147–3150. 10.1073/pnas.91.8.3147 8159717 PMC43532

[acel13958-bib-0050] Zhou, L. , Guo, T. , Meng, L. , Zhang, X. , Tian, Y. , Dai, L. , Niu, X. , Li, Y. , Liu, C. , Chen, G. , Liu, C. , Ke, W. , Zhang, Z. , Bao, A. , & Zhang, Z. (2023). N‐homocysteinylation of alpha‐synuclein promotes its aggregation and neurotoxicity. Aging Cell, 22(3), e13745. 10.1111/acel.13745 36437524 PMC10014048

