# Supplementary material for: Inhibition of abnormal C/EBPβ/α‐Syn signaling pathway through activation of Nrf2 ameliorates Parkinson's disease‐like pathology
Source: Aging Cell. 2023 Aug 23;22(10):e13958. doi: 10.1111/acel.13958 (PMC10577548; doi:10.1111/acel.13958)
Supplement: Supplementary file 1 — Figures S1‐S9 [file ACEL-22-e13958-s001.docx]

**Supplemental Information**

**Supplement**

**Supplement figure 1. Activation of Nrf2 in primary neurons treated with MPP^+^ results in a reduction of C/EBPβ expression in the nucleus**

Treatment with SFN reduced the levels of C/EBPβ expression within the nucleus of primary neurons that have been treated with MPP^+^ (Mean ± SEM, n = 6 per group, one-way ANOVA, **p* < 0.05, and ****p* < 0.001).

**Supplement figure 2. Treatment with C/EBPβ-HDO results in a significant reduction of C/EBPβ expression in SH-SY5Y cells**

**A**: Administration of C/EBPβ-HDO leaded to a reduction in C/EBPβ mRNA expression within SH-SY5Y cells (Mean ± SEM, n = 5 per group, one-way ANOVA, ****p* < 0.001). **B**: Administration of C/EBPβ-HDO leaded to a reduction in C/EBPβ protein expression within SH-SY5Y cells (Mean ± SEM, n = 3 per group, one-way ANOVA, ***p* < 0.01).

**Supplement figure 3. Treatment with C/EBPβ-HDO leads to a significant attenuation of C/EBPβ expression in the nucleus of primary neurons treated with MPP^+^**

Treatment with C/EBPβ-HDO resulted in a significant attenuation of the increase in nuclear C/EBPβ expression within primary neurons treated with MPP^+^ (Mean ± SEM, n = 6 per group, one-way ANOVA, **p* < 0.05, and ****p* < 0.001).

**Supplement figure 4. Glucoraphanin reduces the immunoreactivity of both IBA1 and GFAP in MPTP-treated A53T mice**

Administration of glucoraphanin effectively attenuated the upregulation of both IBA1 and GFAP immunoreactivity within the SNc of MPTP-treated A53T mice (Mean ± SEM, n = 5 per group, one-way ANOVA, ***p* < 0.01 and ****p* < 0.001). Scale bar = 50 μm.

**Supplement figure 5.** **Administration of C/EBPβ-HDO leads to a reduction in C/EBPβ expression within the SNc region of wild-type mice**

**A**: Administration of C/EBPβ-HDO leaded to a reduction in C/EBPβ mRNA expression within the SNc of wild-type mice (Mean ± SEM, n = 5 per group, one-way ANOVA, **p* < 0.05 and ***p* < 0.01). **B**: Administration of C/EBPβ-HDO leaded to a reduction in C/EBPβ protein expression within the SNc of wild-type mice (Mean ± SEM, n = 5 per group, Student’s *t*-test, **p* < 0.05).

**Supplement figure 6. C/EBPβ-HDO reduces the immunoreactivity of both IBA1 and GFAP in MPTP-treated A53T mice**

Administration of C/EBPβ-HDO effectively attenuated the upregulation of both IBA1 and GFAP immunoreactivity within the SNc of MPTP-treated A53T mice (Mean ± SEM, n = 5 per group, one-way ANOVA, **p* < 0.05 and ***p* < 0.01). Scale bar = 50 μm.

**Supplement figure 7. SFN and C/EBPβ-HDO exhibit significant effectiveness in reducing PD pathology in HEK293-α-Syn-YFP cells treated with PFFs**

**A**: Representative images of α-Syn aggregation in HEK293-α-Syn-YFP cells treated with PFFs in the presence or absence of SFN (mean ± SEM, n = 4 per group, Student’s *t*-test, ****p* < 0.01). Green fluorescence spots represent abnormally aggregated α-Syn. Scale bar = 50 μm. **B**: Western blot suggested that administration of SFN significantly ameliorated the downregulation of Nrf2 and the upregulation of C/EBPβ, p-α-Syn, α-Syn, insoluble p-α-Syn and insoluble α-Syn protein expression in HEK293-α-Syn-YFP cells treated with PFFs (mean ± SEM, n = 4 per group, one-way ANOVA, **p* < 0.05, ***p* < 0.01, and ***p* < 0.001). **C**: Representative images of α-Syn aggregation in HEK293-α-Syn-YFP cells treated with PFFs in the presence or absence of C/EBPβ-HDO (mean ± SEM, n = 4 per group, Student’s *t*-test, **p* < 0.05). Green fluorescence spots represent abnormally aggregated α-Syn. Scale bar = 50 μm. **D**: Western blot suggested that administration of C/EBPβ-HDO significantly ameliorated the downregulation of Nrf2 and the upregulation of C/EBPβ, p-α-Syn, α-Syn, insoluble p-α-Syn and insoluble α-Syn protein expression in HEK293-α-Syn cells treated with PFFs (mean ± SEM, n = 4 per group, one-way ANOVA, **p* < 0.05, and ***p* < 0.01).

**Supplement figure 8. C/EBPβ-HDO reduces the immunoreactivity of both IBA1 and GFAP in PFFs-treated A53T mice**

Administration of C/EBPβ-HDO effectively attenuated the upregulation of both IBA1 and GFAP immunoreactivity within the SNc of PFFs-treated A53T mice (Mean ± SEM, n = 5 per group, one-way ANOVA, ***p* < 0.01 and ****p* < 0.001). Scale bar = 50 μm.

**Supplement figure 9. C/EBPβ-HDO reduces the insoluble α-Syn expression in the SNc of PFFs-treated A53T mice**

Administration of C/EBPβ-HDO effectively attenuated the insoluble p-α-Syn and insoluble α-Syn expression within the SNc of PFFs-treated A53T mice (Mean ± SEM, n = 6 per group, one-way ANOVA, **p* < 0.05, ***p* < 0.01, and ****p* < 0.001).
